# Harmful Alcohol Use in Urban Indian Undergraduates: Demographic Patterns and Correlates

**DOI:** 10.7759/cureus.98782

**Published:** 2025-12-09

**Authors:** Beoma Pandey, Pity Koul

**Affiliations:** 1 Sharda School of Nursing Science and Research, Sharda University, Greater Noida, IND

**Keywords:** demographic characteristics, harmful alcohol use, india, parental alcohol use, undergraduate students

## Abstract

Background

Harmful alcohol use among undergraduate students in India is an emerging public health concern, yet research examining the demographic characteristics of students exhibiting harmful use remains limited. Understanding these patterns is crucial for designing early, targeted interventions.

Methods

This analysis draws on data obtained from the control sites of a larger quasi-experimental study. A cross-sectional analysis was conducted using baseline data collected from undergraduate students in two urban co-educational colleges. The Alcohol Use Disorders Identification Test (AUDIT), developed by the World Health Organization, was used to screen alcohol consumption. Students scoring between eight and 19 were classified as harmful alcohol users. Analyses within this subgroup examined associations between AUDIT scores and selected demographic variables using non-parametric statistical tests.

Results

Of the 1,260 students screened, 168 (13.3%) were identified as harmful alcohol users. Within this subgroup, higher AUDIT scores were significantly associated with male gender, being in the final academic year, nuclear family structure, parental alcohol use, and lack of alcohol-related education (p < 0.05). No significant associations were observed with academic performance, type of current residence, or monthly parental income.

Conclusion

Harmful alcohol use was found to cluster within specific demographic groups, underscoring the need for focused screening and tailored preventive approaches on college campuses. Nurse educators and community health nurses are well-positioned to implement early, culturally responsive interventions that could mitigate the progression of harmful drinking among young adults.

## Introduction

Alcohol consumption among young adults, particularly college students, is a growing public health concern in India [1]. This age group, while transitioning from adolescence to early adulthood, gets exposed to academic stress, peer influence, greater freedom of choice, and experimentation that increase their vulnerability to alcohol use [2]. Alcohol continues to be a major risk factor for morbidity and mortality among youth worldwide, contributing to mental health issues, cognitive impairment, violence, and accidental injuries. In 2019, alcohol use was responsible for almost 2.6 million fatalities worldwide [3].

In India, alcohol use among college students has been steadily increasing due to urbanization, greater accessibility, and transforming cultural expectations [4]. It is one of the top five contributing factors for disability-adjusted life years (DALYs) in people aged 15-24 [5]. Research has indicated that the prevalence of alcohol use among undergraduate students in different regions of India ranges from 14% to 30% [6,7]. These emerging trends highlight critical areas for intervention and support in addiction health, underscoring the importance of implementing preventive and targeted strategies within Indian educational institutions.

Drinking habits among college students are frequently linked to low attendance, academic underachievement, dropout risk, and disputes with teachers or peers [8]. Outside of the classroom, harmful alcohol consumption has also been connected to increased vulnerability to anxiety and depression, unsafe sexual behavior, and driving accidents [9].

Despite the growing magnitude of the problem, there remains a lack of research examining how demographic factors vary among individuals exhibiting harmful patterns of alcohol use, a stage where alcohol has started causing detrimental effects on their physical, mental, or social well-being but does not yet meet the requirements for dependence [10,11]. Insights into these demographic variations can support the development of nurse-led, culturally appropriate prevention and screening initiatives [12,13].

A standardized and popular method for screening for alcohol use patterns is the Alcohol Use Disorders Identification Test (AUDIT), created by the World Health Organization (WHO). Scores ranging from eight to 19 indicate harmful alcohol usage [14]. Despite being commonly overlooked, this degree of alcohol use is therapeutically significant because it offers a window of opportunity for timely psychoeducational and community-based interventions by nurses before more serious dependence sets in [15].

Therefore, this study aimed to estimate the prevalence of harmful alcohol use among undergraduate students and to explore demographic variations in AUDIT scores within the harmful-use subgroup using data collected between July and October 2024.

## Materials and methods

Study design and setting

This study is a planned secondary analysis derived from the control arm of a larger quasi-experimental study conducted in four urban co-educational colleges in North India that evaluated the effectiveness of a nurse-led brief psychosocial intervention. For the present analysis, data from two of these colleges, which served as control sites where no intervention was delivered, were examined. As this analysis relies solely on baseline assessments collected at a single time point, it follows a cross-sectional analytical approach to estimate the prevalence of harmful alcohol use among undergraduate students and to explore demographic variations in AUDIT scores within the harmful-use subgroup.

Study population and sampling

The sample size for the broader parent project (which included both intervention and control arms across four colleges) was originally determined using an effect-size-based calculation appropriate for the intervention component. For the present cross-sectional analysis, which uses data from the screened cohort of two colleges (control-arm sites), a prevalence-based justification was applied to confirm the adequacy of the available sample.

Using a 12% prevalence estimate for harmful alcohol use from the pilot assessment conducted during the initial project planning, the minimum required sample was re-estimated using the formula n = (Z² × p(1 − p)) / d², with Z = 1.96 for a 95% confidence level, p = 0.12, and d = 0.05. This yielded a minimum required sample of 162, which was met by the 168 harmful alcohol users identified in the two selected colleges. Thus, the available dataset from the control-arm sites meets both the original effect-size-based requirements of the parent study and the prevalence-based adequacy assessment for the present cross-sectional analysis.

Data collection tools

Two instruments were used for data collection. The first was the self-report version of the WHO AUDIT, which consists of ten questions covering recent alcohol consumption, possible dependence, and negative consequences of drinking, each scored from 0 to 4, with a maximum possible score of 40. Risk levels were categorized as 0-7 (low risk or abstention), 8-19 (high risk or harmful use), and ≥20 (dependence). Only those with scores of eight to 19 were included in the analysis. The second tool was a structured questionnaire developed to capture demographic and alcohol-related characteristics, including age, gender, academic year, current residence, family structure, previous year’s academic performance, monthly parental income, family history of alcohol use, and factors related to alcohol use. This tool was developed through an extensive literature review, expert guidance, and the researcher’s experience. Content validity was established using Lawshe’s Content Validity Ratio (CVR) method, with nine experts rating each item as “essential” or “not essential.” Items scoring below the CVR threshold of 0.78 were revised or excluded. Both tools were pilot-tested on 30 undergraduate students to assess clarity and feasibility.

Data collection procedure

Data collection was conducted between July and October 2024 in designated rooms within the selected colleges after obtaining permission from the Institutional Ethics Committee (reference no. SU/SMS&R/76-A/2023/178; dated September 4, 2023) and the administrative heads of colleges. A proportionate stratified sampling approach was used, with first, second, and third-year classes serving as strata. The required number of students to be screened from each academic year was determined proportionately to that year’s total enrollment. Students were approached during scheduled class hours, and all those present were invited to participate. After the study purpose was explained, written informed consent was obtained from interested students. Within each stratum, consenting students were screened sequentially using the WHO AUDIT until the required quota for that academic year was achieved. The English version of the WHO AUDIT and the structured demographic questionnaire were administered as paper forms. Participants completed the forms independently at their desks, seated apart to ensure privacy. The researcher and trained assistants were available to clarify doubts without influencing responses, and the completed questionnaires were collected immediately in sealed envelopes to maintain confidentiality.

Data analysis

Data were entered into Microsoft Excel (Microsoft Corporation, Redmond, WA) and analyzed using SPSS version 25 (IBM Corp., Armonk, NY). Sociodemographic and alcohol-related variables were summarized using descriptive statistics (frequencies, percentages, means, and standard deviations). Harmful alcohol use was defined as an AUDIT score between eight and 19. Within the harmful-use subgroup, variations in AUDIT scores across demographic categories were examined using non-parametric tests: the Mann-Whitney U test for two-group comparisons and the Kruskal-Wallis H test for variables with more than two categories, as AUDIT scores are ordinal-like and not normally distributed (normality assessed using the Shapiro-Wilk test). For significant Kruskal-Wallis results, post hoc analysis was performed using Dunn’s test with Bonferroni correction (α = 0.017). Point-biserial correlations were calculated for significant dichotomous variables to estimate effect sizes. Statistical significance was set at p < 0.05.

## Results

A total of 168 (13.3%) undergraduate students were identified as harmful alcohol users (AUDIT score eight to 19) from a screened population of 1,260 students across two urban co-educational colleges. The mean AUDIT score among these participants was 9.49 ± 1.67.

Table 1 presents the sociodemographic profile of the identified harmful alcohol users among undergraduate students. The sample comprised 124 (73.8%) male participants, and 97 (57.7%) were in the 20-21 years age bracket. A total of 130 (77.4%) participants belonged to nuclear families, and 70 (41.6%) were in their third academic year. While a large proportion resided in off-campus apartments, paying guest accommodations, or shared housing, only 48 (28.6%) participants lived with their parents. Regarding academic performance, 81 (48.2%) participants had obtained 51-60% marks in the previous year, whereas only 15 (8.9%) had scored above 71%.

**Table 1:** Sociodemographic characteristics of harmful alcohol users among undergraduate students (N = 168) INR: Indian Rupees; PG: paying-guest accommodation

Demographic variable	Frequency (n)	Percentage
Age (in completed years)		
18 years	14	8.3%
19 years	21	12.5%
20 years	53	31.5%
21 years	44	26.2%
>21 years	36	21.4%
Gender		
Male	124	73.8%
Female	44	26.2%
Academic year		
First year	48	28.6%
Second year	50	29.8%
Third year	70	41.6%
Current residence		
Off campus apartment/PG	77	45.8%
Shared house/flat	43	25.6%
Living with parents or guardian	48	28.6%
Family structure		
Nuclear family	130	77.4%
Joint family	38	22.6%
Previous year academic performance		
>71 %	15	8.9%
61-70 %	44	26.2%
51-60 %	81	48.2%
<50 %	28	16.7%
Parental monthly income (INR)		
<30,000	21	12.5%
30,001-60,000	30	17.9%
60,001-1,00,000	78	46.4%
1,00,001-2,00,000	17	10.1%
>2,00,001	22	13.1%

Table 2 shows that a total of 146 (86.9%) participants reported that one or both parents consumed alcohol. Peer pressure was the most common reason for initiating drinking, cited by 86 (51.2%) participants, followed by media influence in 10 (6.0%) and familial influence in seven (4.2%). The initiation of alcohol use most frequently occurred between 15 and 17 years of age, reported by 78 (46.4%) participants, and beer was the preferred beverage for 118 (70.2%). Driving a vehicle while intoxicated was reported by 51 (30.4%) participants, and being a passenger of an intoxicated driver by 40 (23.8%). Only 18 (10.7%) participants had ever received formal alcohol-related education, while 54 (32.1%) were aware of potential legal consequences of alcohol use.

**Table 2:** Alcohol use-related variables of harmful alcohol users among undergraduate students (N = 168) AUDIT: Alcohol Use Disorders Identification Test; ID: identification; PG: paying-guest accommodation

Alcohol use-related variables	Frequency (n)	Percentage
Do either of parents take alcohol		
Yes	146	86.9%
No	22	13.1%
Reason of initiation		
Peer pressure	86	51.2%
Stress	27	16.1%
Influence of media	10	6.0%
Familial influence	7	4.2%
For fun	25	14.9%
Boredom	13	7.7%
Age of initiation		
10 to 12 years	9	5.4%
13 to 14 years	36	21.4%
15 to 17 years	78	46.4%
>18 years	45	26.8%
Preferred beverage		
Beer	118	70.2%
Wine	13	7.7%
Spirits (whiskey/vodka/rum/brandy)	37	22.0%
How do you obtain alcohol		
Purchase myself (with or without fake ID)	122	72.6%
Given by friends/family	38	22.6%
Parties or social events	8	4.8%
Place of consumption		
Social gatherings/parties	35	20.8%
Bars/clubs/restaurants	16	9.5%
At a friend’s place/hostel/PG	117	69.6%
At home	0	0.0%
Ever drove vehicle in drunken state		
Yes	51	30.4%
No	117	69.6%
Have you ever been a passenger to a drunken driver		
Yes	40	23.8 %
No	128	76.2%
Knowledge about legal consequence		
Yes	54	32.1%
No	114	67.9%
Alcohol education received		
Yes	18	10.7%
No	150	89.3%
AUDIT score (mean + SD)	9.49 + 1.67	

Table 3 shows significant differences in AUDIT scores by gender, family structure, and academic year. Males had higher median scores than females (p < 0.001), with a medium effect size (point-biserial r = 0.27). The point-biserial correlation of 0.27 indicated a medium effect size. Similarly, students from nuclear families reported significantly higher AUDIT scores than those from joint families (p = 0.041), with a small effect size (r = 0.14). Significant differences were also observed across academic years (p = 0.001). The mean rank scores were 75.3 for first-year, 71.7 for second-year, and 100.3 for third-year students. Post hoc analysis using Dunn’s test with Bonferroni correction (α = 0.017) revealed that third-year students had significantly higher AUDIT scores compared to both first- and second-year students. No statistically significant associations were found with age, residence type, previous academic performance, or parental income (p > 0.05).

**Table 3:** Association of sociodemographic variables with AUDIT scores among harmful alcohol users (N = 168) H: Kruskal-Wallis H test; U: Mann-Whitney U test Values in parentheses indicate degrees of freedom.

Demographic variable	AUDIT scores		Test	Interpretation
	Mean + SD	Median (IQR)		
Age (in completed years)				
18 years	9.14 ± 1.17	9 (8.25-9)	H(4) = 0.620; p = 0.961	Not significant
19 years	9.76 ± 2.17	9 (9-10)		
20 years	9.58 ± 1.82	9 (8-11)		
21 years	9.43 ± 1.72	9 (8-10)		
>21 years	9.39 ± 1.23	9 (9-10)		
Gender				
Male	9.67 ± 1.73	9 (9-10)	U = 2676.500; p ≤ 0.001	Significant
Female	8.38 ± 0.58	8 (8-9)		
Academic year				
First year	9.08 ± 1.09	9 (8-9)	H(2) = 13.600; p = 0.001	Significant
Second year	9.00 ± 1.08	9 (8-9)		
Third year	10.13 ± 2.11	10 (9-11)		
Current residence				
Off campus apartment/PG	9.47± 1.56	9 (8-10)	H(2) = 0.175; p = 0.916	Not significant
Shared house/flat	9.63 ± 2.12	9 (8-10.5)		
Living with parents or guardian	9.40 ± 1.41	9 (9-10)		
Family structure				
Nuclear family	9.82 ± 2.60	10 (9-11)	U = 1934.000; p = 0.041	Significant
Joint family	8.95 ± 2.52	9 (8-10)		
Previous year academic performance				
>71%	9.60 ± 1.40	9 (9-10.5)	H(3) = 0.610; p = 0.894	Not significant
61-70%	9.43 ± 1.62	9 (8-10)		
51-60%	9.32 ± 1.28	9 (8-10)		
<50%	10.00± 2.62	9 (8-10.25)		
Parental monthly income (INR)				
<30,000	9.86 ± 2.37	9 (9-10)	H(4) = 4.028; p = 0.402	Not significant
30,001-60,000	10.07 ± 2.18	9 (9-11)		
60,001-1,00,000	9.15 ± 1.15	9 (8-10)		
1,00,001-2,00,000	9.71 ± 1.76	9 (8-10)		
>2,00,001	9.36 ± 1.43	9 (8-10)		

Table 4 shows a significant association of AUDIT scores with parental alcohol consumption (p = 0.028), with higher scores among students reporting parental drinking (small effect size, r = 0.13). A significant difference was also observed for alcohol education (p = 0.017), with students who had received any alcohol-related education showing lower scores (r = 0.15), indicating a potential protective effect. Other variables, such as reason for initiation, age of initiation, preferred beverage, method of obtaining alcohol, place of consumption, history of driving after drinking, and awareness of legal consequences, did not show statistically significant associations with AUDIT scores (p > 0.05).

**Table 4:** Association of alcohol related factors with AUDIT scores among harmful alcohol users (N = 168) H: Kruskal-Wallis H test; U: Mann-Whitney U test Values in parentheses indicate degrees of freedom.

Alcohol use-related variable	AUDIT scores		Test	Interpretation
	Mean + SD	Median (IQR)		
Do either of parents take alcohol				
Yes	9.72 ± 1.88	9 (8-11)	U = 1972.000; p = 0.028	Significant
No	9.00 ± 1.80	8 (8-9)		
Reason of initiation				
Peer pressure	9.69 ± 1.88	9 (8-10.75)	H(5) = 3.550; p = 0.615	Not significant
Stress	8.93 ± 1.00	9 (8-9)		
Influence of media	9.20 ± 0.92	9 (9-9.75)		
Familial influence	9.71 ± 1.98	9 (8.5-10.50)		
For fun	9.20 ± 0.82	9 (9-10)		
Boredom	10.00 ± 2.52	9 (9-10)		
Age of initiation				
10 to 12 years	10.00± 1.58	10 (9-10)	H(3) = 3.585; p = 0.310	Not significant
13 to 14 years	9.06 ± 1.07	9 (8-10)		
15 to 17 years	9.60 ± 1.86	9 (8-10)		
>18 years	9.53 ± 1.73	9(9-10)		
Preferred beverage				
Beer	9.53 ± 1.74	9 (9-10)	H(2) = 1.992; p = 0.369	Not significant
Wine	9.92 ± 1.75	10 (8-11)		
Spirits (whiskey/vodka/rum/brandy)	9.22 ± 1.40	9 (8-10)		
How do you obtain alcohol				
Purchase myself (with or without fake ID)	9.37 ± 1.57	9 (8-10)	H(2) = 2.716; p = 0.257	Not significant
Given by friends/family	9.74 ± 1.98	9 (8.25-11)		
Parties or social events	10.12 ± 1.64	9 (9-11.25)		
Place of consumption				
Social gatherings/parties	9.60 ± 2.08	9 (9-10)	H(3) = 0.084; p = 0.959	Not significant
Bars/clubs/restaurants	9.19 ± 1.05	9 (9-9)		
At a friend’s place/hostel/PG	9.50 ± 1.62	9 (8-10)		
At home	NA	NA		
Ever drove vehicle in drunken state				
Yes	9.55± 1.78	9 (9-10)	U = 3075.000; p = 0.743	Not significant
No	9.46 ±1.63	9 (8-10)		
Have you ever been a passenger to a drunken driver				
Yes	9.15 ± 1.12	9 (8-9.25)	U = 2325.000; p = 0.362	Not significant
No	9.59 ± 1.80	9 (8-10)		
Knowledge about legal consequence				
Yes	9.52 ± 1.77	9 (8-10)	U = 2972.000; p = 0.708	Not significant
No	9.47 ± 1.64	9 (9-10)		
Alcohol education received				
Yes	8.00 ± 2.14	8 (8-9)	U = 890.000; p = 0.017	Significant
No	9.12 ± 2.38	9 (8-11)		

## Discussion

The present study highlights the complex interplay of demographic, familial, and educational factors associated with harmful alcohol use among Indian undergraduate students. The findings underscore that harmful drinking is shaped not merely by individual choice but by broader sociocultural and institutional influences that define young adulthood in urban India. This discussion is based on findings derived from the control arm of a larger quasi-experimental study, analyzed here as a planned secondary analysis to explore demographic patterns within the harmful-use subgroup.

The study identified 168 (13.3%) students as harmful alcohol users (AUDIT score 8-19), underscoring an important early intervention window before dependence develops. Harmful use represents a critical stage where timely psychosocial and nurse-led interventions can prevent escalation to alcohol use disorder. Similar findings have been reported in other Indian undergraduate populations, where the prevalence of hazardous or harmful alcohol use ranged from 12-18% [16,17] and in international university samples, prevalence estimates of harmful use were observed at 13-15% [18,19]. These consistent results underscore the importance of implementing preventive strategies in student populations at risk of escalating alcohol use. Consistent with national data and previous research from All India Institute of Medical Sciences (AIIMS) Bhopal, this study found a higher prevalence of harmful alcohol use among male students, with the majority of cases concentrated in the 20- to 21-year age group [20]. This gender gap may reflect not only biological vulnerability but also deeply rooted sociocultural norms that view alcohol use as more acceptable among men. The transitional phase of emerging adulthood, characterized by increased autonomy and experimentation, may further amplify susceptibility to risky drinking behaviors. Living away from parents also emerged as a contextual factor that increases vulnerability, supporting prior findings that lack of parental supervision and social control can facilitate experimentation and regular use [21]. Together, these findings reinforce the need for early preventive interventions that specifically target young men and students living in hostels or rented accommodations.

Peer influence played a significant role in the initiation of alcohol use, echoing trends observed in prior studies among Indian youth [21]. The majority of students reported initiating drinking between the ages of 15 and 17, aligning with national statistics [1,22]. Early initiation of alcohol use is a well-established predictor of later harmful use and dependence, highlighting the urgent need for preventive education in schools and junior colleges before these habits are formed. Beer was the most commonly consumed beverage, a pattern consistent with global data suggesting that affordability, marketing appeal, and perceived social acceptance make it a preferred drink among young adults [23].

Risky behaviors, including drinking and driving, were found to be worryingly frequent even among those aware of legal restrictions, revealing a gap between knowledge and behavioral control. Similar observations have been made by the Ministry of Road Transport and Highways, which identified alcohol-impaired driving as a significant public health issue [24]. This disconnect suggests that punitive approaches alone are insufficient and that sustained, behaviorally informed health education interventions are needed to promote responsible decision-making [25].

The mean AUDIT score among harmful users in this study was 9.49, closely matching the national average reported in previous systematic reviews [1]. As shown in Table 3, significant differences in AUDIT scores were associated with gender, family structure, and year of study. Male students, those belonging to nuclear families, and students in later academic years reported higher mean AUDIT scores. These results reaffirm the gender disparity in harmful drinking patterns previously reported among Indian undergraduates [20,26]. They also highlight the potential protective role of joint family systems, where stronger social cohesion and collective responsibility may serve as buffers against risky behaviors [27]. Conversely, as students progress through academic years, increased autonomy, academic stress, and stronger peer networks may contribute to escalating alcohol consumption [28].

Family environment emerged as a key determinant. Students with parental alcohol use reported significantly higher AUDIT scores, supporting previous findings that parental modeling of drinking behaviors exerts a powerful intergenerational influence [29]. This highlights the importance of family-centered preventive strategies that extend beyond the individual and address family norms and behaviors. On the other hand, participation in structured alcohol education programs was associated with lower AUDIT scores, emphasizing the preventive potential of well-designed, consistent educational interventions [25]. Unfortunately, as national reviews have pointed out, such programs remain inconsistently implemented across Indian educational institutions, limiting their long-term impact [30].

Collectively, the findings indicate that harmful alcohol use among college students varies across specific demographic and alcohol-related factors, particularly gender, family structure, academic year, parental drinking, and prior alcohol-related education. These patterns highlight the influence of family environment and educational exposure on students’ drinking behavior. Interventions aimed at reducing harmful use may therefore benefit from focusing on strengthening preventive education and targeting high-risk subgroups identified in the study. 

Limitations of the study

The cross-sectional design limits causal inference, and the reliance on self-reported data introduces the possibility of under- or over-reporting. Because the analysis was conducted using data originally gathered for another primary objective, the range of variables available for examination was inherently limited. Since the sample included only harmful alcohol users, findings cannot be generalized to abstainers or dependent users. Despite these limitations, the study’s strength lies in its focused examination of an under-researched population, i.e., urban Indian undergraduates, and in its use of standardized tools such as the AUDIT, which enhance comparability with national and international findings.

## Conclusions

Analysis of the control-arm dataset from the parent quasi-experimental study revealed patterns of harmful alcohol use among undergraduate students. Male students, those in later academic years, and individuals from nuclear families demonstrated significantly higher AUDIT scores, indicating greater vulnerability within these groups. Parental alcohol use was associated with higher scores, while participation in alcohol education appeared to have a possible protective effect, as reflected in lower AUDIT scores among those who had previously received such education. This study is among the few in India to focus specifically on harmful alcohol users within a screened undergraduate population and to examine how AUDIT scores vary across demographic and alcohol-related factors in this subgroup, offering context-specific insights that were previously limited.

Based on the observed associations, the implications of this study suggest that preventive strategies in college settings may be strengthened by focusing on subgroups exhibiting higher AUDIT scores, such as male students, students in higher academic years, and those from nuclear families. The association between alcohol education and lower scores indicates the potential value of reinforcing structured educational initiatives. Beyond study-derived implications, broader evidence-based practices may also be relevant, such as implementing structured screening, brief advice sessions, peer-led awareness activities, and individualized counseling within campus health services. Nurses can play a central role in these efforts through systematic screening, educational outreach, and linkage to support services. At the institutional level, colleges may consider developing clear alcohol policies, routine health education sessions, and collaboration with student health services to enhance prevention efforts. Future research should employ longitudinal designs to track changes in alcohol use patterns over time and to evaluate the effectiveness of structured educational and psychosocial interventions. Policy-oriented studies are also needed to examine the implementation of age restrictions, availability controls, and campus-level enforcement strategies. Such evidence would contribute to more robust and culturally relevant alcohol prevention programs for Indian youth.
